# Intervention for School Anxiety and Absenteeism in Children (ISAAC): Mixed-Method Feasibility Study of a Coach-Assisted, Parent-Focused Online Program

**DOI:** 10.1007/s10578-024-01755-6

**Published:** 2024-09-19

**Authors:** Kathryn J. Lester, Brontë McDonald, Alice Tunks, Daniel Michelson

**Affiliations:** 1 School of Psychology, University of Sussex, Brighton, UK; 2 Primary Care and Public Health Department, Brighton and Sussex Medical School, Brighton, UK; 3 Department of Child and Adolescent Psychiatry, Institute of Psychiatry, Psychology and Neuroscience, King’s College London, London, UK; 4 NIHR Maudsley Biomedical Research Centre, South London and Maudsley NHS Foundation Trust and King’s College London, London, UK

**Keywords:** Emotionally-based school avoidance, Online parent intervention, Feasibility, Mixed methods, Primary school-aged children

## Abstract

The aftermath of the Covid-19 pandemic has seen an increase in persistent school absenteeism and Emotionally-Based School Avoidance (EBSA). However, suitable evidence-based psychological interventions are often unavailable. We aimed to assess the feasibility and acceptability of a new parent-focused online program, Intervention for School Anxiety and Absenteeism in Children (ISAAC), which has been co-designed with parents and practitioners. This exploratory mixed-method study recruited participants from three schools in southern England, enrolling N = 9 parents for whom a child, aged 5–11 years, was experiencing signs of EBSA. The intervention consisted of three web-based psychoeducational modules respectively addressing parental stress, accommodating parenting behaviors, and communication with school staff. Module completion was assisted by weekly calls with a non-specialist “coach.” Feasibility was measured using indicators of retention, module participation, overall program completion and coaching fidelity. Acceptability was assessed using semi-structured interviews, module ratings and written qualitative feedback. We also explored baseline-post change in parent-reported measures of children’s school avoidance, absences, anxiety, parental stress, accommodating parenting behaviors, and quality of parent-school communication. Overall, the intervention was feasible to deliver to parents with six (67%) participants completing the full intervention. Participants found the intervention acceptable across thematic domains of affective attitude, burden, coherence, self-efficacy and perceived effectiveness. Participants particularly appreciated the coach’s support. We observed small to moderate reductions in school avoidance behaviours (*d* with Hedges correction = 0.36), child anxiety (*d* with Hedges correction = 0.33) and accommodating behaviours (*d* with Hedges correction = 0.44) at the post timepoint compared to baseline. In conclusion, ISAAC shows early promise with the potential to deliver scalable online support for families affected by emerging EBSA. Future research should move toward establishing effectiveness in a randomized controlled trial.

Children with emotionally-based school avoidance (EBSA) experience significant emotional distress and avoidance related to school attendance [1]. Prevalence rates for EBSA are generally estimated to be between 1–5% of school-aged children based on studies conducted in a range of school systems worldwide [2]. However, an accurate picture of the prevalence of EBSA in England following the Covid-19 pandemic is not known, although we might reasonably expect it to be higher given the sharp rise in school absenteeism. In England, persistent absences in primary school (missing > 10% of school sessions) have increased following the COVID-19 pandemic from 11.2% in 2018 to 20.9% in 2022 [3]. Emotional factors including heightened anxiety and distress about attending school have been identified as playing an important part in this upward trend [4, 5].

EBSA peaks in the secondary school years but often emerges during primary schooling, suggesting the need for early intervention before patterns of poor attendance become entrenched [6, 7]. EBSA can have deep and long-lasting negative effects on children’s development and life prospects (see [8] for a review). The school absences associated with EBSA are consistently associated with poorer academic achievement (e.g. [9, 10]), increased likelihood of school dropout [11], compromised social skills and peer relationships [8, 12, 13], increased mental health and behavioural problems and relationship difficulties [8, 14] and poorer employment prospects in later life [15]. Parents also report significant distress and frustration in managing EBSA [16].

In England, absences caused by EBSA are commonly recorded as unauthorized triggering punitive measures such as parental warnings and financial penalties [17]. Such statutory responses lack evidence and are usually viewed as ineffective and antagonistic by parents [18, 19]. A recent review of school-based attendance interventions found little compelling evidence that family incentives/disincentives, mentoring, social and emotional skills programmes, behaviour interventions or provision of meals and/or extracurricular activities improve attendance. More promising evidence was identified for programmes that centred on parental engagement and communication, and responsive and targeted programmes attending to one or more barriers to attendance [20]. For the relatively few families who schools refer to specialist psychological support, cognitive behavioural therapy (CBT) can be an effective approach to tackle EBSA [2]. CBT interventions often involve direct work with children to strengthen coping strategies and practice exposure tasks, with conjoint parenting sessions to address “family accommodations” (e.g., repeated reassurance) that can maintain anxious avoidance of school in the longer-term [21, 22]. However, specialist CBT can be difficult to access, particularly in the context of increased referral thresholds and capacity limitations in child and adolescent mental health services (CAMHS) since the COVID-19 pandemic [23, 24]. Furthermore, there is equivocal evidence that specialist CBT improves key functional outcomes including attendance [25]. This may be due to the relative lack of attention given to wider systemic influences on school absenteeism.

In a previous qualitative study conducted with 39 families affected by EBSA, we investigated risk factors for EBSA and explored viable intervention strategies in the post-pandemic context [19]. We subsequently conducted workshops with parents and professionals to co-design a scalable parent-focused intervention for emerging EBSA called Intervention for School Anxiety and Absenteeism in Children [ISAAC, 26]. In this work, we drew on existing socio–ecological frameworks that acknowledge that risk factors for EBSA are multi-faceted, operate at school, family and individual levels [27–29] and have been amplified by the Covid-19 pandemic [30]. We were also informed by research indicating that parental engagement is key to successfully addressing EBSA [16, 20].

Our qualitative research revealed a parent population demoralised and demotivated by treatment failures, service gaps and sometimes critical attitudes from education staff. Parents identified key risk factors for EBSA including difficulties managing their own frustration and worries, not knowing how best to respond when children get upset about attending school, and feeling misunderstood and judged by school staff [19]. Co-design activities highlighted preferences for a brief, online format that could be accessed flexibly to fit around parents’ daily lives [26]. ISAAC therefore comprises three web-based psychoeducation modules centred on (1) helping parents with managing their own stress and wellbeing through identifying indicators of stress and teaching evidence-based stress management techniques (e.g. breathing exercises, self-care activities,(2) teaching positive parenting strategies (e.g. confidence-building statements to reduce the use of accommodating behaviours (e.g. repeated reassurances, modifying routines to avoid anxiety triggers that attempt to prevent or reduce child distress and (3) building more positive relationships with school staff through the teaching of solution-focused strategies to improve communication. Content is delivered through animated videos, “real-world” vignettes, brief reflective activities and reinforced through a home learning task which provides the opportunity to put into practice the skills parents have learned from the intervention in their daily lives. The goal of the intervention is to reduce distress related to school and improve attendance through the theorized mechanisms of improved parental stress management and self-efficacy, a reduction in well-intentioned accommodating parenting behaviours that can inadvertently maintain child anxiety and avoidance of school and the building of more trusting, collaborative relationships with school staff.

Co-design participants also favoured a guided approach that incorporated non-judgmental, interpersonal support from a “coach” to facilitate use of online content. Coaching is often included in online interventions due to its positive impact on participant engagement and outcomes [31–33]. However, in line with evidence for community-based ‘task-sharing’ approaches ([34] i.e., where behavioural healthcare tasks are delivered by lay people and other non-specialists), this support need not be provided by highly-qualified and relatively scarce therapists. ISAAC has therefore been designed to include brief support from a non-specialist coach at the outset of the intervention and following the completion of each module.

The current study aimed to evaluate the feasibility and acceptability of ISAAC using a pre-post design. The aims of the study were:

- To examine the feasibility of recruiting, retaining and engaging parents with a child experiencing signs of EBSA and delivering ISAAC as a guided online intervention
- To investigate whether parents consider the intervention to be acceptable in terms of affective attitude, burden, coherence, self-efficacy and perceived effectiveness
- To provide preliminary data on the potential benefits associated with receiving ISAAC on parent-reported measures of children’s school avoidance, absences, child anxiety, perceived stress, accommodating behaviors and the extent to which parents trust and communicate with their child’s school.

## Methods

### Design

We used a mixed-method, pre-post design that integrated data from quantitative feasibility indicators, participant-reported outcome measures, and semi-structured qualitative interviews. Ethical approval was obtained from the Research Ethics Committee at the University of Sussex (Reference:bm333/9).

### Participant Eligibility Criteria and Recruitment

Eligible participants were parental caregivers (“parents”) of a child aged 5–11 years, enrolled in a mainstream primary school, and experiencing emotional distress associated with school attendance. Given our focus on early intervention for EBSA, we did not specifically screen for anxiety nor impose a predetermined absenteeism criterion. Instead, we took a pragmatic and inclusive approach by stipulating only that the child should be experiencing significant distress when anticipating and/or actually attending school. This was determined by parental report with parents asked to indicate via a screening question when indicating their interest in participation whether their child had experienced anxiety about attending school in the current academic year. This was then confirmed verbally with parents during a brief onboarding call. Children with neurodevelopmental comorbidities or other “special educational needs” [35] were not excluded. Parents needed access to an internet-enabled device, and to read and speak English as required to engage with the content of the intervention. Exclusion criteria included not being resident in the county of Sussex, England and the child not being registered to attend a mainstream school.

Participant referrals were generated in collaboration with a school-based mental health service provided by local authority staff in Sussex. Staff in this service distributed information to four partner schools, out of which three schools shared recruitment materials with parents. Materials were passed directly to parents of children with known difficulties related to EBSA, as well as being shared more widely within whole-school communications.

### Measures

#### Feasibility Data

We conceptualized feasibility as the extent to which the intervention could be successfully delivered as intended [36]. Specific feasibility indicators related to retention (number of participants who completed all three modules as a proportion of those who started the intervention), module participation (mean number of modules completed and mean number/duration of coaching sessions attended by those who started the intervention); time taken to complete the intervention; and coaching fidelity. Following a method used in other guided intervention studies with a facilitating “coach” [37], fidelity was assessed by an independent rater who scored all recorded coaching sessions using a 3-point scale (0 = not completed, 1 = partially completed, 2 = fully completed) across six items: (1) engaging the participant and setting the agenda; (2) reviewing progress with module content; (3) answering queries about module content; (4) reviewing the homework learning task; (5) planning the next module; and (6) concluding the session. Scores across the six components were summed for each module with a higher score indicating greater coaching fidelity.

#### Preliminary Outcome Data

Six parent-reported measures were used to assess child- and parent-focused outcomes at baseline (prior to the on-boarding call) and at the post assessment timepoint approximately 6-weeks later (see Table 1 for measure descriptions).

**Table 1:** Parent-reported outcome measures administered at baseline and 6-week post-assessment timepoints

	Outcome	Measure
Child outcomes	School avoidance	The School Refusal Assessment Scale-Revised-Parent version [38] is a 24-item scale that assesses current school avoidance behaviours. Items are scored from 0 (*never*) to 6 (*always*). The sum of all items is calculated with higher scores indicating greater school avoidance symptoms and behaviours. The SRAS-R has been validated in numerous countries reporting adequate internal consistency and satisfactory test–retest reliability [39]
	School absences	Parents reported the number of days on which their child was absent from school due to emotionally-based avoidance in the preceding 6-weeks (not counting school closures/excused absences)
	Anxiety	The Spence Children’s Anxiety Scale Short-Form [40] is an 8-item scale that assesses current symptoms of child anxiety. Items are scored from 0 (*never*) to 6 (*always*). The sum of all items is calculated, with higher scores indicating more severe anxiety. The short-form version has demonstrated acceptable to good internal consistency, good convergent/divergent validity and is able to discriminate between clinic and community-referred samples [40]
Parent outcomes	Stress	The Perceived Stress Scale (PSS; [41]) is a 10-item scale that assesses attributions for life events as being unpredictable, uncontrollable and overloading over the previous month. Items are scored from 0 (*never*) to 4 (*very often*). The sum of all items is calculated with higher scores indicating greater stress. The PSS-10 has been very widely-used and has good internal consistency and satisfactory construct validity [42, 43]
	Accommodating behaviours	Family Accommodation Scale-Anxiety [FASA, 44] is a 13-item scale that assesses the frequency with which family members currently demonstrate accommodating behaviours such as reassurance, participation in avoidance and modifying routines. Items are scored from 0 (*never)* to 4 (*daily*). The sum of all items is calculated with higher scores indicating more extensive accommodating behaviours. The FASA has strong psychometric properties including convergent/divergent validity and satisfactory test–retest reliability [45]
	Home-school communication	The Trust/Communication subscale of the Parent Engagement in Early Childhood Education [PEECE, 46] includes 10-items designed to assess the extent to which parents trust and communicate with their child’s school. Items are scored from 1 (*never true*) to 4 (*always true*). The sum of all items is calculated with higher scores indicating more trust and communication with the school. The PEECE Trust/Communication subscale has good internal consistency and convergent/divergent validity [46]

#### Acceptability Data

We developed a semi-structured topic guide that was informed by the Theoretical Framework of Acceptability [47]. Questions addressed affective attitude (how participants felt about the intervention), burden (the amount of effort that is required to take part in the intervention); coherence (the extent to which participants understood the intervention and how it worked); self-efficacy (the extent to which the participant felt capable of performing the behaviours required by the intervention); and perceived effectiveness (the extent to which the intervention was perceived as achieving its purpose).

Upon completion of each module, participants rated their module experience on a 5-point scale marked by smiling emoji faces ranging from 1 (*very unhappy*) to 5 (*very happy*). At the post-assessment timepoint parents provided an overall rating of the perceived helpfulness of the intervention in improving their child’s attendance and lowering their child’s emotional difficulties about school on a scale from 0 (*not helpful at all*) to 4 (*extremely helpful*). They were then invited to provide brief qualitative written feedback on the one or two best things about the intervention and any areas for improvement.

### Procedures

#### Enrolment

Interested parents accessed a URL that linked to an information sheet and consent form. The enrolment window was open for six weeks (24th April–5th June 2023). The researcher confirmed participant eligibility via a brief telephone call, during which they also shared a URL for the participant to access the baseline assessment measures and booked a subsequent onboarding session (see intervention description below) with consented eligible participants. Participants were asked to complete their baseline assessment prior to attending their onboarding call).

#### Intervention

**Fig. 1:**
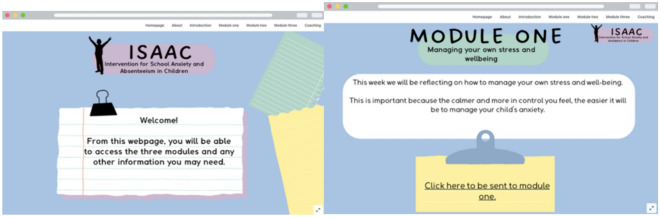
Example screenshots from ISAAC website

Table 2 provides an overview of the ISAAC program according to the Template for Intervention Description and Replication [TIDieR, 48]. Briefly, the intervention comprised three online modules covering strategies to manage parent stress and wellbeing, guidance on modifying parental accommodating behaviors that may inadvertently maintain school-related anxiety and avoidance; and solution-focused strategies for communicating effectively with school staff. Modules were hosted on Qualtrics and accessed through a password-protected website (see Fig. 1).

**Table 2:** Description of ISAAC using TIDieR framework

WHY Goal, rationale and content	The goal is to reduce children’s school-related anxiety and associated absenteeism by targeting three modifiable risk factors: parental stress, family accommodation of child anxiety; and poor home-school communication Improvements in these focal areas are achieved through: self-care strategies to increase parental wellbeing and self-efficacy; parenting strategies to change accommodating behavioural patterns that may inadvertently maintain children’s distress and avoidance of school; and solution-focused strategies to improve home-school communication
WHAT Materials	An online webpage provides links to three modules corresponding to each of the respective risk factors mentioned above. Each module comprises of four videos that encompass psychoeducation on the weekly topic and relevant vignettes, self-completed reflective tasks, and a homework learning task
WHAT Procedures	The videos and written vignettes provide psychoeducation related to module-specific adaptive strategies. The reflective tasks provide opportunities to link these strategies to the participant’s own circumstances. The homework learning task encourages applied use in real-life situations. A coach introduces the intervention via an “onboarding” session and meets with the participant after each module to clarify any questions related to module content; to plan and review homework; and to troubleshoot technical or other barriers to learning and generalising adaptive strategies
WHO PROVIDED Expertise and background of provider	A coach provides remote guidance according to a structured coaching protocol. The coach is unconnected to the child’s school and does not have a specialist background in mental health care. Supervision (approx. 30 min every 1–2 weeks) is provided by a Clinical Psychologist to reflect on session process issues and factors affecting fidelity to the coaching protocol
HOW Modes of delivery	The intervention is provided to individual parent participants. Three online modules are accessed via a password protected webpage. Participants interact remotely with a coach via video-conferencing software, telephone and/or text
WHERE Location(s) where the intervention occurred	Participants access the intervention in their own homes and/or any other convenient locations
WHEN and HOW MUCH Number of sessions, their schedule, and their duration	Following an initial onboarding session, modules are designed to be completed on a weekly basis followed by corresponding coaching sessions. There is some flexibility to extend this time if needed, such that the total duration can vary from 3–6 week. Module videos and reflective tasks can be completed within 30–60 min; coaching sessions last approximately 20–30 min

**Fig. 2:**
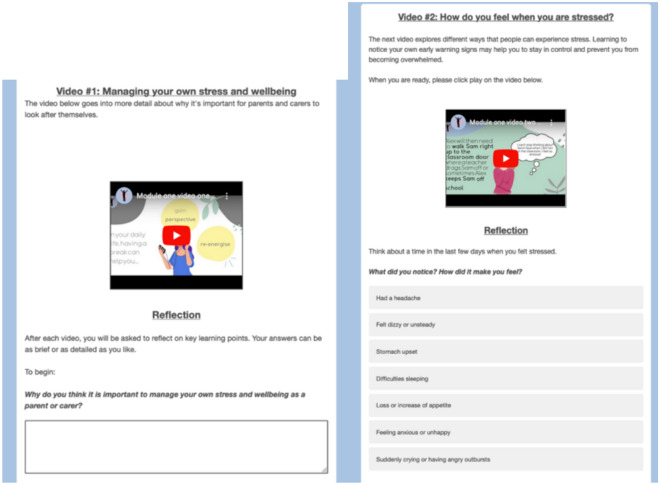
Example screenshots of Module 1 content

Core content was presented through animated videos which included psychoeducational content and related vignettes depicting real-world examples. Psychoeducational content was consolidated through the completion of brief reflective activities, for example participants were invited to identify and list out accommodations that occur in their family, and to try out a relaxation technique. Each module concluded with goal setting for a homework learning task to complete offline (e.g., practising one or more chosen stress management strategies, producing a plan for a meeting with school). Screen-shots from Module 1 are provided as illustrative examples in Fig. 2.

An initial “onboarding” session (lasting approximately 20–30 min) was provided over a voice or video call by an assigned coach (the second author). The coach was a non-specialist with a background in mental health research (registered as a PhD researcher in Psychology) and previous teaching experience. Prior to this study they completed training in safeguarding and active listening. They received approximately weekly supervision from a Clinical Psychologist (DM) who was also available should any urgent concerns arise during a coaching session.

All onboarding calls were completed between 17th May–6th June 2023. The purpose was to orient participants to the intervention’s aims, structure and activities. This meeting also included a tutorial about how to access the web platform and explained the role of the coach.

It was recommended that participants should complete one module each week, following a fixed order, but with the flexibility to extend the timeframe if necessary. Each module was followed by a coaching session, lasting approximately 20–30 min, which was conducted via voice or video call according to a written coaching protocol. Coaching aimed to enhance engagement and outcomes by clarifying/expanding on key concepts and adaptive strategies; encouraging completion of the homework learning task; troubleshooting technical or other barriers to completing the intervention modules and applying the content to everyday situations.

Ad-hoc text-message support was available from the coach during normal working hours, with a one-day response time. The coach also sent two reminders (via text and email) to complete each module and in advance of scheduled coaching sessions. These routine messages also provided the option to reschedule sessions if required.

#### Assessments

Outcome measures were administered via an online survey in Qualtrics with baseline assessments (including a demographic proforma) completed before the on-boarding call and post-assessments completed approximately six weeks later (M interval = 52 days, range = 42–70 days). Nine parents completed both their baseline and post-assessments and received a £20 e-voucher as reimbursement for their time. Participants were also invited to take part in an optional qualitative interview about the acceptability of the intervention, for which they were offered a further £20 e-voucher. Five participants (all of whom had completed three modules) participated in this qualitative interview. Interviews lasted for 40–60 min and were conducted over voice or video call by an independent researcher who was not involved in intervention development or delivery (AT).

### Analysis

Quantitative feasibility indicators and parent-reported outcome measures were summarised descriptively using frequency counts, proportions, means, and SDs. We report effect sizes using Cohen’s *d* with Hedges g correction. Qualitative data were analyzed using a thematic framework approach [49]. Analysis proceeded in a step-wise fashion, moving back and forth through steps when required. To begin, the second author (BM, a PhD student in Psychology) independently read all interview transcripts to familiarise themselves with the data. A preliminary coding framework was then prepared deductively by the second author from the research questions, drawing on an established acceptability framework for healthcare interventions [47] and applied to the first few transcripts. The analysis then proceeded by organizing preliminary codes into categories within a working analytical framework through team-based discussion, with these codes/categories then applied to all subsequent transcripts. We iteratively and inductively adapted the framework to take account of any new insights derived from the data. Finally, themes were brought together and integrated with quantitative data on individual module experience ratings, overall intervention perceived helpfulness ratings and qualitative written feedback about the strengths of the intervention and areas for improvement to map and interpret the dataset as a whole using a mixed-methods framework matrix [50]. This allowed us to explore potential convergence and divergence between quantitative and qualitative data to provide a detailed picture of intervention acceptability.

The analysis was led by the second author (BM), who is a PhD researcher in Psychology and former primary school teacher. They approached the analysis from a critical realist perspective, whilst being mindful of their own assumptions and experiences from their teaching background [51]. Weekly supervisory discussions with senior co-authors (KL, D.Phil in Psychiatry and DM, DClinPsy Doctorate in Clinical Psychology) were used to develop and refine the analytical framework and critically examine assumptions and interpretations.

## Results

### Feasibility

**Fig. 3:**
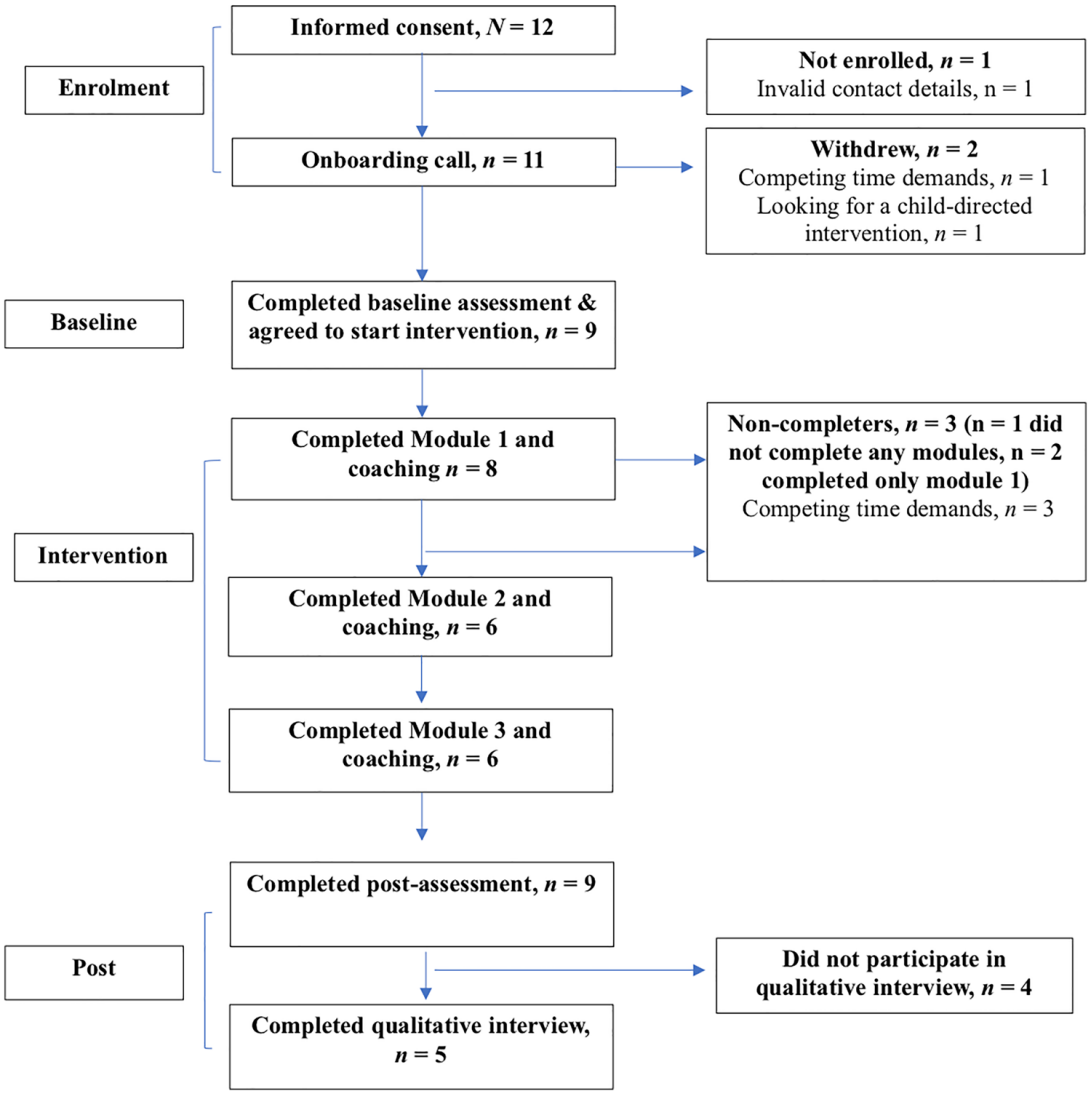
Participant study flow

N = 12 parents provided informed consent (see Fig. 3 for participant flow). Due to the indirect recruitment methods, it was not possible to determine how many children attended the schools who received study information and within those schools how many parents were informed about the study relative to the number consenting. Out of the 12 parents who consented at the outset, one parent did not respond to further contact attempts, two parents did not proceed beyond the onboarding call, and nine parents participated in the intervention and completed paired baseline and post outcome assessments. Table 3 summarizes the demographic characteristics of these nine participants. Out of those nine participants, one participant (11%) started the first module but did not complete it; two participants (22%) completed the first module and corresponding coaching session only, and six participants (67%) completed all three modules and corresponding coaching sessions. Parents who completed the full program did so in M = 34.33 days (time from onboarding call to the third coaching session: range = 28–49 days, SD = 7.84).

**Table 3:** Participant characteristics (N = 9)

	n/(%) or Mean (sd)
Parent characteristics	
Gender	
Female	8 (89%)
Male	1 (11%)
Ethnicity	
White British	7 (78%)
White Irish	1 (11%)
White another background	1 (11%)
Household income	
< £29,999	2 (22%)
£30,000-£49,999	2 (22%)
> £50,000	5 (56%)
Child characteristics	
Gender	
Female	5 (56%)
Male	4 (44%)
Eligibility for Free School Meals	
Yes	3 (33%)
No	6 (67%)
Prefer not to say	1 (11%)
Child has an Education Health and Care Plan	
Yes	1 (11%)
No	6 (67%)
Prefer not to say	1 (11%)
Child has additional needs without Education Health and Care Plan	
Yes (Autism, PDA, ADHD)	1 (11%)
No	8 (89%)
Support currently provided to family by	
School Mental Health Service	2 (22%)
Learning mentor	2 (22%)
SENCO^†^	1 (11%)
None	6 (67%)
Age (years, mean (sd))	8.00 (1.94)

Twelve parents consented at the outset, of which nine started the intervention and completed paired outcome assessments

^†^Special educational needs co-ordinator

Coaching sessions were a similar duration for each module (Module 1: M = 22.02 min, SD = 11.78, range 15–29 min; Module 2: M = 21.27, mins, SD = 0.17, range 15–27 min; Module 3: M = 22.31 min, SD = 0.25, range 16–32 min). One parent opted for coaching via voice call with the remainder conducted via video call. The coach delivered the intervention with fidelity for each module (Module 1: M = 11.29 (out of a maximum score of 12), SD = 1.25; Module 2: M = 11.33, SD = 0.82; Module 3: M = 11.40, SD = 0.89).

### Preliminary Outcomes

As shown in Table 4, school avoidance symptoms and behaviours (SRAS) decreased from baseline (M = 61.07, SD = 15.09) to post-assessment (M = 53.56, SD = 14.37) with a small effect size (Cohen’s *d* with Hedges correction = 0.36). A similar pattern of change was observed for child anxiety (SCAS-P-8; baseline: M = 11.56, SD = 3.43; post: M = 11.00, SD = 2.83; Cohen’s *d* with Hedges correction = 0.33). Parent-reported absences increased marginally from baseline to post-assessment (baseline: M = 1.11 days, SD = 1.45; post: M = 1.33 days, SD = 3.64; Cohen’s *d* with Hedges correction =− 0.06), although this finding was primarily driven by a large increase in number of days absent for a single child with the remaining children showing either no change or a reduction in parent-reported absences.

**Table 4:** Preliminary parent-reported outcomes at baseline and post-assessment timepoints (n = 9)

Outcome measure	Mean (SD)		Effect Size of Change Between Baseline and Post (Cohen’s *d* with Hedges g Correction) (95% Confidence Intervals)
	Baseline	Post	
School avoidance behaviours and symptoms (SRAS-R-P)	61.07 (15.09)	53.56 (14.37)	0.36 (− 0.26, 0.97)
School absences (days)	1.11 (1.45)	1.33 (3.64)	− 0.06 (− 0.65, 0.54)
Child anxiety (SCAS)	11.56 (3.43)	11.00 (2.83)	0.33 (− 0.29, 0.93)
Perceived stress (PSS)	27.00 (6.18)	25.89 (3.95)	0.12 (− 0.48, 0.71)
Accommodating parenting behaviours (FASA)	31.89 (7.90)	28.22 (7.05)	0.44 (− 0.20, 1.05)
Home-school communication (PEECE)	24.33 (3.94)	23.56 (5.05)	0.54 (− 0.12, 1.17)

*SRAS* school refusal assessment scale-revised, *PSS* perceived stress scale, *SCAS* spence children’s anxiety scale, *FASA* family accommodation scale—anxiety, *PEECE* the parent engagement in early childhood education

Accommodating parenting behaviors were reduced at post-assessment compared to baseline with a small to medium effect size (FASA; baseline: M = 31.89, SD = 7.90; post: M = 28.22, SD = 7.05; Cohen’s *d* with Hedges correction = 0.44). A smaller effect was observed for perceived stress scores (PSS; baseline: M = 27.00, SD = 6.18, post: M = 25.89, SD = 3.95; Cohen’s *d* with Hedges correction = 0.12). The extent to which parents trust and communicated with their child’s school diminished from baseline to post-timepoint, which was not anticipated, (PEECE; baseline: M = 24.33, SD = 3.94; post: M = 23.56, SD = 5.05; Cohen’s *d* with Hedges correction = 0.54). All of the observed effect sizes had confidence intervals that crossed zero.

### Acceptability

#### Affective Attitude

Overall, parents were positively inclined towards the intervention. “It was quite nice. I even suggested that there could be more modules because I wanted to learn more.” (Parent 10) Individual module experience ratings suggested that all modules were rated highly and did not reveal clear differences between them in terms of participant’s experience (Module 1: M = 4.6, SD = 0.55; Module 2: M = 4.75, SD = 0.45; Module 3: M = 4.5, SD = 0.82, out of a maximum score of 5).

Material in Module 1 (concerned with stress management and self-care) helped to create the psychological conditions needed for participants to implement the more child-focused strategies in the subsequent modules.

“So the self-care—I think what I really liked was the way it was set up, so starting with the parents. That was really good and just that whole thing about not blaming yourself, that this probably isn’t due to you, I think that was really important to hear that first of all. Because if you’re always beating yourself up about that and feeling guilty, then it’s hard to move forward really. So that was really good.” (Parent 11)

In Module 2, parents appreciated psychoeducation about the anxiety-avoidance cycle and concrete suggestions about how to break this cycle by modifying their accommodating behaviors.

“That one I did find helpful because I started to understand about enabling because I, you know, will do whatever I can to, sort of, support the kids, but then thinking about it from that point of view, you know, actually sometimes it isn’t actually helpful to, you know, allow everything.” (Parent 6)

Parents noted that Module 3 introduced concrete practical solutions they could draw on when communicating with school staff, and engendered a more constructive perspective, in which the school could be viewed (to an extent) as a collaborative partner rather than a source of antagonism.

“What was nice about this one, it was very standalone and it was something that was pretty—I hadn’t really put thought into this…. and yes, it was very helpful to just have this space to think about how to do it and what you would want out of it. And the idea of getting stuff in writing.” (Parent 4)

“I suppose my husband and I had become quite negative about the school, but not in front of our son and other parents as well, we were very careful not to do that. But even between us we were like, “Right, we need to draw a line underneath that now and move on.” (Parent 11)

Parents looked forward to their coaching sessions and valued the coach’s attentiveness, active listening skills, and personalized approach, all of which fostered a strong rapport. Parents also valued the coach’s independence from their child’s school, which encouraged more candid dialogue.

“[The coach] is a very open person...I could always ask about something if I had a worry, so it was nice that I could just talk about it, and she didn’t straightaway say, ‘Back to the topic.’ She was really a good listener” (Parent 10)

“Great to have somebody to talk to, but the same person to talk to each week, and I felt that she really got to know us and, well, me and the situation really quickly. I didn’t just feel like a number, it felt really personal.” (Parent 11)

#### Burden

Parents found the intervention manageable and sufficiently flexible to accommodate within their daily lives. They appreciated the options to reschedule coaching sessions and complete the modules in multiple sittings rather than in a single attempt. They acknowledged challenges associated with finding time for module completion, however, parents regarded the investment as worthwhile. “You do have to make time for these kinds of things if you want changes in your lives, in your struggles. So, it’s not a sacrifice…it’s been a helpful thing.” (Parent 2).

Interviewees indicated that extending the intervention timeframe to allow for a longer module completion period would improve manageability further and provide more time to practice skills in daily life.

“What I would say as a reflection on everything as a whole is I found—the bit that was a bit tricky was that you had to do your reflection, do the task, write yourself a task and then do the task all in the same week before you had your coaching session.” (Parent 4)

They also recommended adding an extra coaching session to each module, which could then be used to discuss module content and plan homework learning tasks together, followed by a further session to review homework prior to moving on to the next module. Some parents also expressed a desire for their partners to take part jointly. It was additionally recommended that a concise summary of each module should be made available as a helpful reminder of key points or for partners who cannot complete the full course due to other commitments.

“I’m just wondering whether the core, sort of, values of the course could be put into like a little really, you know, like two lines or something like that then people … you could like have it stuck on your fridge.” (Parent 6)

#### Coherence

Parents indicated that the onboarding call provided a useful orientation to the intervention. They found the intervention structure cohesive and appreciated that modules 2 and 3 began by briefly recapping the previous modules key messages before progressing to the videos and reflective tasks for the current module. “I think it was designed well. It refers back to the previous module, isn’t it, at the beginning. After you watch the next set of videos. It’s designed well, I think.” (Parent 2).

Parents particularly praised the video animations as a clear and visually-appealing method for presenting information, particularly for parents with reading difficulties but also recommended that they be displayed in a larger format. Parent 6 stated “*…*the videos do definitely, you know, make it really clear because I suppose I think sometimes you feel a bit like you’re not exactly sure what you’re being asked to do, but yes that made it very easy” while Parent 4 described the module structure and content as “very accessible, easy to wrap your head round…there was no problems with understanding what was being asked.”

#### Self-Efficacy

The reflective tasks and home learning activities within each module enhanced parents’ understanding of the psychoeducational video content. Parents underscored the pivotal role of the coach in supporting their self-efficacy to participate in the intervention, attributing the coach’s role in accountability, and adherence to set timelines as essential for their progress. The coach’s guidance was also particularly valuable in translating psychoeducational content into practical guidance for the homework learning tasks. Parents indicated that without the coaching element, implementation of guidance into their everyday lives would have been more challenging and adherence lower. Parent 6 described the positive impact of the coaching on their depth of engagement and ability to put into practice the skills they had learned, “The other things you could possibly do on your own, like the research side of it, but I don’t think I would have actually implemented any of the stuff without that coaching session.” Likewise, parent 2 described the impact on their adherence to completing the modules, “I think without the coaching session, it would have been just too easy to not do the modules. It was just a good way of reflecting back on what we’ve been learning through the modules.”

#### Perceived Effectiveness

At the post-assessment timepoint, parents indicated that the intervention was ‘somewhat helpful’ for reducing their child’s anxiety about school (M = 2.22, SD = 0.97). Additionally, parents felt, on average, that the intervention was ‘somewhat helpful’ for improving their child’s school attendance (M = 2.00, SD = 1.12). All interviewees qualitatively described the intervention as being helpful and some explicitly indicated that they would recommend it to other parents whose children were experiencing signs of EBSA, “Yes, I think I would recommend it to someone, if it was running for my wife I’d say, Yes, give it a go, it’s a useful way to think about things.” (Parent 4); “Yes, definitely, I have recommended it to my friend already.” (Parent 6).

Beyond child-focused outcomes, parents reflected that the skills learned in Module 1 contributed to calmer household routines, which in turn led to improvements in family relationships.

“… thinking about what can be done differently, to support the morning routines of everyone. So, it doesn’t happen every day but laying out the kids’ clothes or even my clothes in the morning. Having the warm packed lunches prepared the night before. These kinds of things are really helpful in the morning and everyone is just less stressed and a lot happier.” (Parent 2)

They also described being more confident in their ability to communicate with their child’s school.

“Your program really has helped me to understand I can turn to school and I have ways of communicating with the school.” (Parent 4) and “The time to reflect on the communication with the school was really helpful and the idea of what information you do really need and the pathway of communication between both. Because as good as the school is, I don’t think their communication is particularly good. So that I thought probably for me was the most helpful bit.” (Parent 11)

Parents were particularly positive about the benefits of using ‘confidence-building statements’ (Module 2), which offered a positive alternative to their previous accommodations. However, parents of children with SEN/neurodevelopmental comorbidities conveyed challenges in distinguishing between accommodating behaviors and reasonable adjustments for their child’s wider constellation of needs. While participants recognised the intervention as a useful starting point, they indicated that sustained changes would require more substantial assistance.

“I spoke with [the coach] about this, it can be hard to figure out what was an accommodation and what would be a reasonable adjustment. There’s some stuff that you have to say, ‘Well, this is because she’s autistic and we have to adjust our lives to make this comfortable’… But her fighting over refusing to brush her teeth and us brushing her teeth for her, is probably an accommodation rather than a reasonable adjustment. So once I’d got my head around that, I thought it was a bit more helpful.” (Parent 4)

“I do think that a single session for looking at behavioural change, I don’t think it is enough to really institute those behaviours. And the middle bit is just a bit too short to make a lasting change from.” (Parent 6)

Other suggested improvements to Module 2 included use of more real-life examples (backed by testimonials from fellow parents) about how to substitute adaptive parenting strategies in place of accommodations; and offering materials that could be shared directly with children. In addition, parents suggested that the timing of the study during the late Summer term might have limited the potential benefits of ISAAC by restricting the time available for implementation of new self-care, parenting and home-school communication strategies. Parents recommended that it would be advantageous to offer ISAAC at the start of the academic year and/or in a responsive way when needed and suggested by doing so it may have a greater impact on attendance.

“I wonder if it would be better to do the program at an earlier time in the year. I think children who are struggling to attend school would be prone to miss the last couple of weeks of the year at school due to school fatigue and exhaustion.” (Parent 11)

“…but if I did the course a bit earlier, I would have done things differently” (Parent 2).

“Attendance wise it didn’t really seem to make a difference but we’re just not sure whether because at the time when the programme was run, I sort of feel if it had been run earlier in the year perhaps it would have had more of an impact.” (Parent 6).

## Discussion

We examined the feasibility, and acceptability of ISAAC, a novel parent-focused online intervention that is intended as an early intervention for primary-school aged children experiencing early signs of EBSA. Our findings indicate that ISAAC is acceptable to parents, and feasible to deliver as a web-based parent-led intervention with remote guidance provided by a non-specialist coach. It was feasible to recruit parents in collaboration with existing school mental health services and to retain them as the majority of parents successfully completed the intervention. In terms of acceptability, participants valued the online modular format, which could be accessed flexibly and with minimal practical barriers to participation. Coaching offered parents a supportive space in which to discuss their children’s difficulties and their own worries, as well as facilitating completion of homework learning tasks. We found preliminary evidence for reductions in parent-reported measures of accommodating behaviours, school avoidance behaviours and symptoms and child anxiety. Estimated effect sizes were in the small to medium range. However, in the absence of a control group, any reported changes cannot be reliably attributed to the intervention. Parents felt that the intervention would have been more effective if it had been available closer to the start of the academic year. There were also indications that the intervention may require refinements for parents of children with special educational needs/neurodevelopmental comorbidities.

This study adds to the literature on guided online interventions provided in contexts where children and their families might otherwise struggle to access specialist mental health services. Participants dedicated approximately 3–4 h of their time to accessing the intervention modules and coaching, which was experienced as a worthwhile investment of time. This aligns with the typical expectations for low-intensity CBT therapy, which usually involves six hours or less of contact time [52]. Despite the brief and flexible nature of the intervention, it is noteworthy that some participants did not complete the full intervention, citing competing time demands as the primary reason for this. Future research could explore whether the current intervention specification could be made yet more efficient, scalable and flexible by enabling participants to pick from a menu of modules according to their own goals and circumstances or by extending the timescales for intervention completion.

The coach emerged as a key influence on participants’ engagement with ISAAC. Prior research has shown that self-directed online interventions typically experience challenges with poor adherence and drop-out [53], which can be mitigated through synchronous support [54], especially under conditions of “supportive accountability”. The latter refers to conditions where “human support increases adherence through accountability to a coach who is seen as trustworthy, benevolent, and having expertise” [55]. This concept helps to explain the pivotal role played by the ISAAC coach. In line with task-sharing approaches, our coach was a non-specialist without extensive formal training or clinical qualifications in mental health care. However, they were nevertheless perceived by participants as being both credible and caring, which are characteristics that increase responsiveness to accountability demands [56]. Any future efforts to implement ISAAC on a larger-scale should be attentive to such interpersonal qualities, both in the selection and training of prospective coaches. Future research could explore the potential to train peer volunteers as intervention coaches. A parent-to-parent delivery model may not only reduce implementation costs but could also enhance credibility and sustainability of the intervention.

This small, uncontrolled feasibility study was not designed to test the effectiveness of ISAAC. However, we cautiously note that small to medium sized effects in the expected direction were observed on parent-reported outcomes of accommodating parenting behaviours, children’s school avoidance (encompassing a variety of avoidant behaviors and symptoms) and child anxiety. These effects are corroborated by qualitative interviews and quantitative feedback in which parents rated the intervention as somewhat helpful in reducing their child’s anxiety about school and identified Module 2 (focused on reducing accommodating behaviors) as being particularly impactful. Other research has highlighted the importance of family accommodations in maintaining child anxiety in general and school avoidance specifically [21, 22]. It is important to note that it is not possible to attribute the changes on parent-report measures to the intervention due to the lack of a control group. Approximately a third of the children were receiving some form of additional support on top of receiving ISAAC. It is possible that this may, in part, explain the improvements observed. The intervention was also delivered in the last half term of schooling before the Summer holidays. Anxiety and avoidance of school may have dissipated naturally as children approached the end of the Summer term as this often tends to be a period of less academic intensity in many schools.

We also note that our multi-item measure of school avoidance behaviours and symptoms appeared to be a more sensitive measure of change than actual absences. This discrepancy may partly reflect a floor effect, such that absolute absence rates were very low at baseline. We did not apply a specific eligibility criterion around absenteeism, consistent with our interest in early intervention. It is plausible that larger effects would be observed on measures of absence in a sample experiencing more persistent absenteeism and distress about attending school. Elsewhere, the small to negligible effects observed for other outcomes and the unexpected finding that parent-reported trust and communication with school decreased may be an indication that parents had insufficient time to put intervention strategies into practice, given the relatively short duration before post-assessments and the scheduling of the study during the final term of the academic year. This line of reasoning is supported by qualitative findings, wherein participants felt that greater benefits would accrue from having access to ISAAC earlier in the year allowing for more time to put into practice the guidance and strategies learned.

### Strengths and Limitations

A notable strength of this study is its use of mixed methods to achieve a nuanced and contextualized understanding of ISAAC’s feasibility, and acceptability [57]. This research design enhances the study’s capacity to thoroughly explore the intervention’s feasibility and offers clear guidance for future iterations of the intervention.

Nevertheless, the current study has some notable limitations. First, effect sizes should be interpreted with caution due to the small sample size, wide confidence intervals and lack of a control group. Second, all outcomes in the study were parent-reported. Third, qualitative interview data were not available from parents who did not complete the intervention despite being invited to participate in an exit interview. Future research would benefit from multi-informant measures of key outcomes, a more objective approach to measuring attendance using official school data, and purposive sampling of non-completers. Fourth, study participation was open to parents of all genders, but mothers predominantly took part. The study also lacked representation from families from diverse ethnic, socioeconomic and geographical backgrounds. Future evaluations should include a more diverse range of participants.

Finally, while parents of children with SEN/neurodevelopmental disorders were favorably disposed toward the intervention and found it helpful, they felt that more comprehensive support that acknowledged and addressed the complexities of parenting a child with SEN/developmental disorders, and which addressed some of the school-based factors [58] that contribute to EBSA in this group was needed. Consequently, future iterations of ISAAC should explore whether the inclusion of additional SEN-specific material is warranted.

### Conclusion

ISAAC was co-designed with parents and relevant professionals to meet the need for a scalable early intervention that addresses modifiable risk factors for EBSA that are prioritised by parents. Our findings suggest that a coach-supported modular format constitutes a feasible and acceptable approach for delivering efficient, online support to families where a child, aged 5–11 years, is struggling with early signs of EBSA. The detailed feedback from parents in this study will be instrumental in refining ISAAC prior to further testing [45].

## Summary

This study investigated the feasibility and acceptability of a novel parent-focused online program, Intervention for School Anxiety and Absenteeism in Children (ISAAC) that is intended as an early intervention for primary-school aged children experiencing EBSA. ISAAC consists of three web-based psychoeducational modules addressing parental stress, accommodating parenting behaviours and communication with school staff alongside regular support from a non-specialist coach. We recruited nine parents for whom a child, aged 5–11 years was experiencing signs of EBSA and provided them with access to ISAAC. The study assessed feasibility using indicators of retention, module participation, overall program completion and coaching fidelity. Acceptability was assessed using semi-structured interviews, module ratings and written qualitative feedback. We also explored baseline-post change in parent-reported measures of children’s school avoidance behaviours and symptoms, absences, child anxiety, perceived stress, accommodating parenting behaviors, and quality of parent-school communication. Results showed that ISAAC is acceptable to parents, and feasible to deliver as a web-based parent-led intervention with remote guidance provided by a non-specialist coach. Parents particularly valued the brief, flexible format and non-judgemental support from the coach. We observed small to medium-sized reductions in parent-reported measures of accommodating behaviours, school avoidance behaviours and symptoms and child anxiety. Overall, ISAAC shows early promise with the potential to deliver scalable online support for families affected by emerging EBSA.

## Data Availability

The detailed coding framework and data analysis used in this study is available on request from the corresponding author. Participants did not provide permission to share raw data (i.e., unprocessed transcripts) outside of the core research team.
